# Genome of the extinct Gotland cattle breed

**DOI:** 10.1186/s12864-025-12382-3

**Published:** 2025-12-03

**Authors:** Martin Johnsson, Anna M. Johansson

**Affiliations:** Department of Animal Biosciences, Swedish University of Agricultural Sciences, Box 7023, Uppsala, 750 07 Sweden

**Keywords:** Cattle, Historical DNA, Extinct breeds

## Abstract

**Background:**

The extinct cattle breed Gotland cattle lived on the island of Gotland in the Baltic Sea until the beginning of the 1950s. We sequenced the genomes of two Gotland cattle isolated from skulls from a local museum on Gotland.

**Results:**

The depth of coverage was 2.7X and 3.3X, respectively, with a breadth of coverage of 85% and 89%. Based on coverage of the sex chromosomes, both animals appeared to be female. We detected 19 million single nucleotide variants and 2.8 million indels in the joint dataset of Gotland cattle jointly called with modern Swedish cattle. In a principal component analysis, the two Gotland cattle placed the closest to Swedish Red cattle, rather than among the southern or northern traditional breeds. In terms of mitochondrial haplotypes, they were similar to clusters of related haplotypes involving multiple other breeds, including Swedish Mountain cattle, Swedish Red Polled and several Finnish cattle breeds.

**Conclusions:**

In summary, our results suggest that Gotland cattle were genetically closer to the ancestors of Swedish Red cattle than to the extant traditional Swedish breeds.

**Supplementary Information:**

The online version contains supplementary material available at 10.1186/s12864-025-12382-3.

## Background

**Fig. 1:**
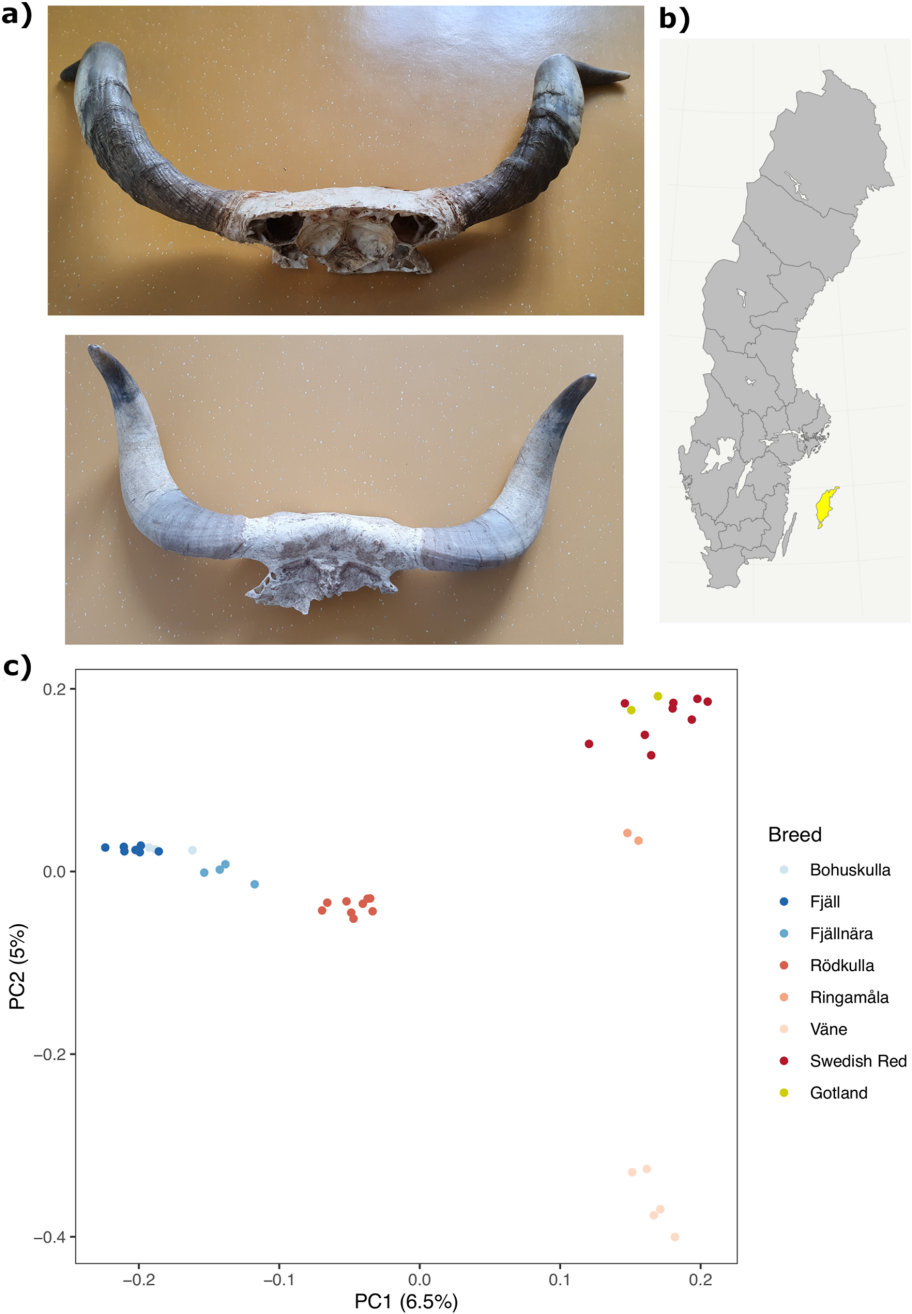
**a** Photographs of the two Gotland cattle skulls sampled for this study. **b** Map of Sweden indicating the island of Gotland in yellow. The map was made with the ggplot2 and swemaps2 R packages. For a map that shows the location of origin of other Swedish breeds, we refer to Fig. 1 of [2]. **c** Scatterplot of the first two principal components showing the two Gotland cattle samples (in yellow) in relation to other Swedish local breeds. The horizontal axis shows the first principal component, and the vertical axis shows the second principal component, with variances explained in parentheses. The colour of the dots indicates breed

The extinct cattle breed Gotland cattle (Gotlandsko in Swedish) lived on the island of Gotland in the Baltic Sea until the beginning of the 1950 s [1]. Gotland cattle were of small size and often had yellow coat colour and big horns. We sequenced the genomes of two Gotland cattle, based on bone samples from skulls. The skulls originate from a local museum in Viklau in the middle of Gotland and are shown in Fig. 1.

The origin of Gotland cattle is not clearly known, but possible routes of migration of cattle to Gotland include both from east and south across the Baltic Sea. There was probably also gene flow from the mainland, such as from native cattle from the Småland region. At times, the Småland and Gotland breeds were considered contiguous. Before the decline, Hallander [1] gives a typical census size for the breed of around 20,000 animals. By the end of the 19th century, Gotland cattle were largely replaced by red cattle, and during the last decades, crossing with red cattle was also common. The last Gotland cattle bull was approved for breeding in 1909.

## Methods

### Sample preparation and sequencing

DNA isolation and library preparation were performed under ancient DNA conditions at the Ancient DNA Unit at SciLifeLab, Uppsala (Uppsala University). Before cutting, bones were cleaned with water and ethanol, and after cutting, bone samples were decontaminated by UV treatment and washed with bleach solution. DNA was isolated with a silica-based protocol described by [3] based on [4]. The samples were first incubated in 0.5 M EDTA and then with 0.4 mg/ml proteinase K. The extraction buffer contained 1 M urea instead of sodium dodecyl sulphate. After extraction, samples were purified with the MinElute PCR Purification kit (Qiagen). First, one library was prepared for each sample, pooled at equimolar ratios and sequenced on one lane of an Illumina NovaSeq SP flow cell, generating paired-end 2 × 150 bp reads. After analysis of the first libraries, sample 1 showed higher number of bovine reads. The percentage of bovine DNA was 62% in sample 1 and 39% in sample 2, indicating a larger contamination of other DNA in sample 2. Therefore, three additional libraries were prepared from sample 1 and six from sample 2, for a total of nine libraries that were pooled at equimolar ratios and sequenced. Sequencing was performed at the SciLifeLab SNP&SEQ Technology Platform (Uppsala University).

### Mapping and processing of alignments

The reads were mapped and preprocessed using the SciLifeLab Ancient DNA unit using their bioinformatics pipeline. Reads were trimmed with Cutadapt v. 2.3 [5] to remove adapter sequences and low-quality bases with a base quality less than 15. The paired-end reads were merged with FLASh v. 1.2.11 [6] requiring a minimum overlap of 11 bp. Reads were mapped to the ARS-UCD1.2 reference genome [7], including the Y chromosome from the Btau5.0.1 assembly with bwa aln [8] with parameters -l 16,500 -n 0.01 -o 2. Alignments were merged into one set of aligned reads per sample using SAMtools v.1.10 [9]. After merging, the alignment was processed by removing PCR duplicates with FilterUniqSAMCons_cc.py [10], removing reads shorter than 35 bp, and removing reads with less than 90% identity to the reference genome. The resulting depth and breadth of coverage of the cattle genome was assessed with BEDTools v. 2.29.2 [11]. The depth of coverage on sex chromosomes was inspected to infer the sex of the animals.

### Variant calling

We analysed the two Gotland cattle samples together with a dataset of 39 Swedish cattle, consisting of the 30 cattle of Swedish traditional breeds previously studied by [12] and 9 Swedish Red cattle sequenced at the department for use in the 1000 Bull genomes project (available in the European Nucleotide archive at accession number PRJEB76973). After the read processing described above, which was specific to the historic Gotland cattle samples, mapping and variant calling were performed in the same way for historical and recent samples.

We used the Sarek workflow version 2.7.1 implemented in Nextflow [13, 14] to perform variant calling. Sarek runs the bwa mem [15] aligner followed by GATK [16, 17] germline variant calling workflow, including marking of duplicates with Picard (http://broadinstitute.github.io/picard/) and base quality score recalibration followed by the HaplotypeCaller to produce GVCF files containing genotype likelihoods for each sample, which were then used for joint variant calling.

We combined the GVCF files from the two Gotland cattle samples with the other Swedish cattle samples in the same GenomicsDB dataset, and ran joint variant calling with GATK (version 4.2.6.1) GenotypeGVCFs. After variant calling, we separated the single nucleotide variant calls and insertions/deletions, and performed hard filtering using standard thresholds QD < 2.0, QUAL < 30.0, SOR > 3.0, FS > 60.0, MQ < 40.0, MQRankSum < −12.5, ReadPosRankSum < −8.0 for single nucleotide variants and QD < 2.0, QUAL < 30.0, FS > 200.0, ReadPosRankSum < −20.0 for indels.

In order to summarise the genotype distribution and reads supporting genotypes in the Gotland cattle samples, we used bcftools version 1.9 [9] to extract the genotypes and allelic read counts for each variant.

### Principal component analysis and model-based clustering

We used Plink version 1.90 [18] to perform principal component analysis on biallelic single nucleotide variants from the full dataset of Gotland cattle combined with the 39 modern cattle. Because the outcome of principal component analysis is sensitive to the composition of the sample included, we performed several principal component analyses with different subsets of the Swedish cattle samples. In one analysis, we excluded the two Gotland cattle samples. In another, we excluded Swedish Red cattle, because it is the biggest group and also the only major commercial breed with an intense breeding program. In three successive analyses, we randomly subsampled the breeds down to include a maximum of two, five or seven individuals from each group. We also applied model-based clustering with ADMIXTURE version 1.3.0 [19] to biallelic single nucleotide variants. The number of ancestral populations (K) ranged from 2 to 8 (i.e., the number of breeds in the dataset).

### Potentially functional variants in candidate genes

We selected a list of candidate genes (Table S1) known to be associated with genetically simple traits in cattle, such as coat colour and casein protein variants and major quantitative trait loci for complex traits from the OMIA database [20]. We used the Ensembl Variant Effect Predictor version 107 [21] to detect potentially loss-of-function variants (variants classified by VEP as “HIGH” impact) and potential missense variants in these genes, including variants where the alternative allele was observed in at least one of the two Gotland cattle samples.

### Mitochondrial DNA analysis

We extracted consensus mitochondrial DNA sequences from the two Gotland cattle samples and 30 local Swedish cattle belonging to traditional breeds using bcftools. We extracted all variants called on the mitochondrial genome, normalized the insertion/deletions using bcftools norm, and then used bcftools consensus to generate one consensus sequence from each individual, using the ALT allele calls at each variant.

We compared the mitochondrial DNA from the two Gotland cattle samples to whole mitochondrial sequences from the 30 local Swedish cattle, as previously used by [12]. We also compared them to 108 Nordic and Baltic cattle mitochondrial D-loop sequences from GenBank from [22], including Swedish, Danish, Finnish and Estonian samples. We aligned sequences using Clustal Omega [23] and created median-joining haplotype networks with PopART [24]. We used EMBOSS Seqret [25] to convert between Clustal and Nexus file formats, and the R package ape [26] to trim the D-loop alignment to exclude basepairs missing from all the Kantanen et al. samples.

## Results

We detected 19 million single nucleotide variants and 2.8 million insertions/deletions in the dataset of Gotland cattle jointly called with 39 modern Swedish cattle. In the Gotland cattle samples, 15% and 9.3% of the single nucleotide variants had missing values, which can be compared to an average missingness of 1.3% in the modern samples. The depth of coverage was 2.7X and 3.3X, respectively, with a breadth of coverage of 85% and 89%. Based on coverage of the sex chromosomes (Figs. S1-S4) where the X chromosome had similar coverage as the autosomes, both animals appeared to be female. The depth of coverage of the mitochondrial genome was 195X and 316X, respectively, with a breadth of coverage of 100%.

In a principal component analysis, the two Gotland cattle samples were similar to each other and placed the closest to Swedish Red (SRB) cattle, rather than among the southern or northern traditional breeds. Fig. 1 shows scores on the first two principal components derived from biallelic single nucleotide variants. Both in terms of the first and the second principal component, the two Gotland cattle samples had similar scores to the Swedish Red Cattle. In terms of the first principal component, the second closest breed was Ringamåla cattle. When performing the principal component analysis with subsets of the data as a robustness check (Fig. S5), these qualitative patterns were preserved. The two Gotland cattle samples were the closest to Swedish Red cattle even when breeds were subsampled to have more even sample sizes, and were genetically closest to Ringamåla cattle when Red Cattle were excluded from the analysis.

Model-based ancestry estimation with ADMIXTURE (Figure S6) gave rise to a similar clustering of animals as the principal component analysis, where runs with low numbers of hypothetical ancestral populations (K) separated the northern from southern breeds. Gotland cattle were consistently placed among the southern breeds and clustered together with Swedish Red animals until K = 7.

The two Gotland cattle samples carried a few new potentially functional variants in genes known to be involved in monogenic traits. Supplementary table S1 shows the potential loss-of-function variants and Supplemental table S2 the missense variants detected in known candidate genes with the observed allele count in the modern breeds. There were three potential loss-of-function variants detected in the two Gotland cattle samples: one variant causing loss of the start codon in the *MITF* gene (22:g.31650963T > C), one frameshift insertion/deletion in the *MC1R* gene (18:g.14705685del), and one splice donor variant in the *CSN1S2* gene (6:g.85530668G > A). The *MC1R* frameshift variant was common in all breeds, whereas the *MITF* variant was fixed or near fixed in both traditional breeds and Swedish Red cattle. There were 26 potential missense variants in candidate genes in the two Gotland cattle samples, including one in the *KIT* gene and two in the *TYRP1* gene.

**Fig. 2:**
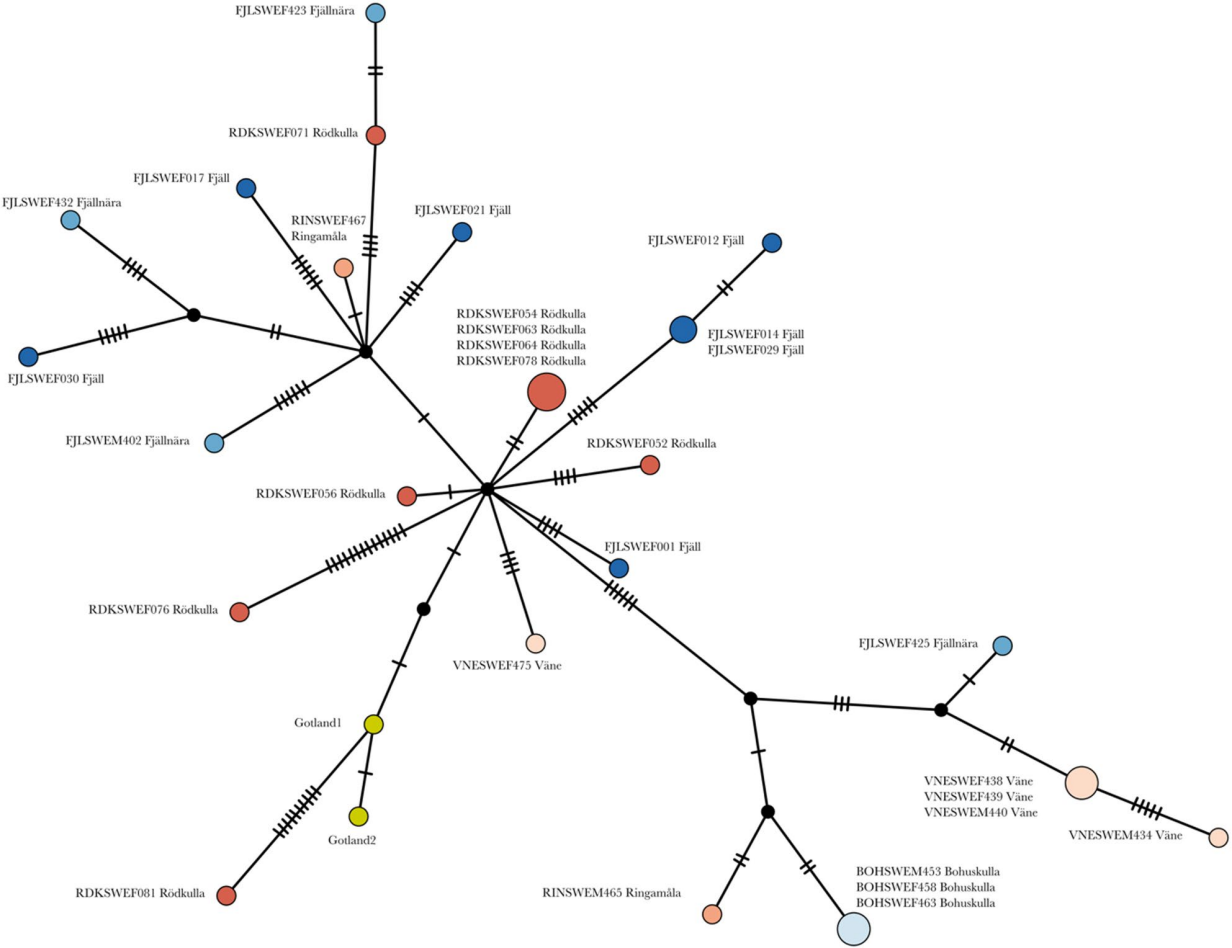
Mitochondrial DNA haplotype network of the two Gotland cattle samples (in yellow) in relation to mitochondrial DNA from other Swedish local breeds

Figure 2 and Fig. S7 show median joining networks of mitochondrial haplotypes from the two Gotland cattle samples compared to the 30 local Swedish cattle, and to Nordic and Baltic D-loop sequences [22]. The Gotland cattle haplotypes were similar, separated by one variant located outside of the D-loop region. They were not identical to any of the other mitochondrial haplotypes, neither among the 30 local Swedish cattle or the D-loop sequences. However, they were similar to clusters of related haplotypes involving multiple other breeds, including Swedish Mountain cattle (Fjällko), Swedish Red Polled (Rödkulla), and multiple Finnish cattle breeds.

## Discussion

Our results suggest that Gotland cattle were genetically dissimilar to the extant traditional Swedish breeds and place it closer to the ancestors of Swedish Red cattle. This may be due to crossing with Swedish Red cattle, which is reported to have been common during the last decades of the breed [1], or to deeper shared ancestry since the Swedish Red breed has an admixed origin that includes southern Swedish native red cattle. The fact that the mitochondrial haplotypes were not identical to any other Nordic and Baltic breeds suggests that the maternal origin may be from older types of cattle on Gotland. Bulls from the ancestors of Swedish Red cattle on mainland Sweden may have been brought to Gotland for breeding, making the Gotland cattle similar to Swedish red cattle on the nuclear DNA. Such a discrepancy between the mitochondrial and the nuclear DNA ancestry has also been observed for some present day traditional Swedish cattle breeds [12].

Our results identified a few potentially functional variants in candidate genes. These variants, however, were not unique to Gotland cattle, as they were shared with and often common in other Swedish breeds. The other variants we detect in pigmentation-related genes *MITF*,* KIT* and *TYRP1* are not among the known causative variants described in the OMIA database. The frameshift variant in *MC1R*, previously known as the “e” allele (OMIA variant ID: 1762) that causes a recessive red phenotype [27], was carried in a heterozygous state by both Gotland cattle and was observed in all the other Swedish breeds in our study, as previously reported by [12]. As such, we have not detected novel functional variation in the Gotland cattle samples. We note, however, that several known causative variants in such genes as the *KIT* gene (observed for example in Swedish Mountain cattle [28–30]) are structural variants. Structural variants are difficult to reliably detect in short read data, and even more so with historical DNA.

Historical and ancient DNA analyses are limited by low sequencing coverage due to DNA breakdown and damage. There is a risk of high missingness allelic drop out, where heterozygous variants cannot be distinguished since only one allele is sequenced. On the other hand, the samples in this study were from the mid-20th century and thus relatively recent compared to ancient DNA, and missing genotype rate was around 10–15%, leaving many variants for the principal component analysis. Especially for the mitochondrial analyses, the coverage was high.

## Supplementary Information

Supplementary Table S1

Supplementary Table S2

Supplementary Figures

## Data Availability

The sequence data for the Gotland cattle are available at accession number PRJEB60559, for the 30 Swedish cattle of extant local breeds are available at accession number PRJEB60564, and for the 9 Swedish Red cattle are available at accession number PRJEB76973 in the European Nucleotide Archive.
