# Supplementary material for: Genome of the extinct Gotland cattle breed
Source: BMC Genomics. 2025 Dec 3;26:1093. doi: 10.1186/s12864-025-12382-3 (PMC12690854; doi:10.1186/s12864-025-12382-3)
Supplement: Supplementary file 1 — Supplementary Table S1 [file 12864_2025_12382_MOESM1_ESM.pdf]

Supplementary Table S1. Potential loss-of-function variants detected in candidate genes in Gotland cattle, showing chromosome, position, reference genome and alternate alleles, gene symbol, genotype in the two Gotland cattle samples (coded as 00 for homozygous reference, 01 for called heterozygote and 11 for homozygous alternate), and alternate allele counts out of total number of chromosomes for the other breeds. Note that due to the risk of allelic drop-out, homozygotes may not have been accurately distinguished from heterozygotes in the Gotland cattle samples.

Chr	Position	REF	ALT	Gene	Gotlandsko1	Gotlandsko2	Bohuskulla	Fjäll	Fjällnära	Rödkulla	Ringamålako	Väneko	SRB	Potential consequences
6	85530668	G	A	*CSN1S2*	01	00	0 / 6	0 / 14	1 / 8	1 / 18	0 / 4	0 / 10	0 / 16	XM_024993017.1 splice_donor_variant
18	14705684	CG	C	*MC1R*	01	01	2 / 6	6 / 14	4 / 8	8 / 18	3 / 4	6 / 10	7 / 16	ENSBTAG00000023731 frameshift_variant
22	31650963	T	C	*MITF*	11	11	6 / 6	14 / 14	8 / 8	18 / 18	4 / 4	10 / 10	15 / 16	ENSBTAG00000006679 start_lost
