# Supplementary material for: Genome of the extinct Gotland cattle breed
Source: BMC Genomics. 2025 Dec 3;26:1093. doi: 10.1186/s12864-025-12382-3 (PMC12690854; doi:10.1186/s12864-025-12382-3)
Supplement: Supplementary file 2 — Supplementary Table S2 [file 12864_2025_12382_MOESM2_ESM.pdf]

Supplemental Table S2. Missense variants detected in candidate genes in Gotland cattle, showing chromosome, position, reference genome and alternate alleles, gene symbol, genotype in the two Gotland cattle samples (coded as 00 for homozygous reference, 01 for heterozygote and 11 for homozygous alternate), alternate allele counts out of total number of chromosomes for the other breeds, and potential consequences for protein sequence as predicted by VEP. When multiple splice variants lead to proteins with the same amino acid substitution in the same position, the table only gives one transcript accession. Note that due to the risk of allelic drop-out, homozygotes may not have been accurately distinguished from heterozygotes in the Gotland cattle samples.

Chr	Position	REF	ALT	gene	Gotlandsko1	Gotlandsko2	Bohuskulla	Fjäll	Fjällnära	Rödkulla	Ringamålako	Väneko	SRB	Potential consequences
3	116981583	A	G	*MLPH*	11	..	2 / 6	4 / 14	4 / 8	9 / 18	3 / 4	2 / 10	3 / 16	ENSBTAP00000000837:p.Met113Val
3	116981637	C	A	*MLPH*	11	00	0 / 6	4 / 14	4 / 8	5 / 18	3 / 4	2 / 10	2 / 16	ENSBTAP00000000837:p.His131Asn
3	116981638	A	G	*MLPH*	11	00	2 / 6	4 / 14	4 / 8	9 / 18	3 / 4	2 / 10	5 / 16	ENSBTAP00000000837:p.His131Arg
3	116981649	G	A	*MLPH*	11	00	0 / 6	4 / 14	1 / 8	4 / 18	0 / 4	1 / 10	2 / 14	ENSBTAP00000000837:p.Ala135Thr
3	116996878	T	C	*MLPH*	11	00	0 / 6	4 / 14	2 / 8	4 / 18	0 / 4	1 / 10	4 / 16	ENSBTAP00000058971:p.Met387Thr, ENSBTAP00000000837:p.Met393Thr, XP_024845194.1:p.Met400Thr, XP_024845193.1:p.Met402Thr
3	116997197	G	A	*MLPH*	00	01	0 / 6	4 / 14	2 / 8	4 / 18	0 / 4	1 / 10	5 / 16	ENSBTAP00000058971:p.Arg419Gln, ENSBTAP00000000837:p.Arg425Gln, XP_024845194.1:p.Arg432Gln, XP_024845193.1:p.Arg434Gln
3	117002401	A	G	*MLPH*	11	00	0 / 6	4 / 14	2 / 8	4 / 18	0 / 4	1 / 10	3 / 16	ENSBTAP00000058971:p.Thr565Ala, ENSBTAP00000000837:p.Thr571Ala, XP_024845194.1:p.Thr578Ala, XP_024845193.1:p.Thr580Ala
4	27814937	G	T	*TWIST1*	11	11	6 / 6	11 / 14	8 / 8	16 / 18	4 / 4	7 / 10	14 / 16	XP_024846774.1:p.Trp64Leu
6	70214244	T	C	*KIT*	11	11	6 / 6	10 / 14	8 / 8	18 / 18	4 / 4	10 / 10	16 / 16	ENSBTAP00000003498:p.Met258Thr
6	85451132	C	G	*CSN2*	11	11	6 / 6	12 / 14	8 / 8	17 / 18	4 / 4	10 / 10	14 / 16	XP_015327157.2:p.Arg129Ser, ENSBTAP00000003409:p.Arg172Ser
6	85451298	T	G	*CSN2*	00	11	2 / 6	7 / 14	5 / 8	7 / 18	3 / 4	5 / 10	9 / 16	ENSBTAP00000003409:p.His117Pro, XP_015327157.2:p.His74Pro
6	85536434	G	T	*CSN1S2*	01	11	0 / 6	0 / 14	0 / 8	2 / 18	0 / 4	0 / 10	1 / 16	XP_015327143.1:p.Glu65Asp, ENSBTAP00000006590:p.Glu74Asp
6	85656736	T	C	*CSN3*	01	00	0 / 6	3 / 14	4 / 8	9 / 18	4 / 4	8 / 10	1 / 14	ENSBTAP00000065313:p.Ile146Thr, ENSBTAP00000028685:p.Ile157Thr
6	85656772	C	A	*CSN3*	01	01	0 / 6	3 / 14	4 / 8	9 / 18	4 / 4	8 / 10	2 / 12	ENSBTAP00000065313:p.Ala158Asp, ENSBTAP00000028685:p.Ala169Asp
8	31639069	C	T	*TYRP1*	00	01	1 / 6	8 / 14	3 / 8	14 / 18	4 / 4	4 / 10	11 / 16	ENSBTAP00000027945:p.Glu329Lys, XP_015327928.2:p.Glu362Lys
8	31647440	G	C	*TYRP1*	00	01	0 / 6	0 / 14	0 / 8	0 / 18	0 / 4	3 / 10	0 / 16	ENSBTAP00000027945:p.Thr112Ser, XP_015327928.2:p.Thr145Ser
11	103257950	G	A	*LBG*	11	00	3 / 6	4 / 14	0 / 8	3 / 18	2 / 4	8 / 10	7 / 16	ENSBTAP00000019538:p.Gly80Asp
11	103259232	C	T	*LBG*	11	11	3 / 6	4 / 14	0 / 8	3 / 18	2 / 4	8 / 10	3 / 16	ENSBTAP00000019538:p.Ala134Val
11	103260219	T	C	*LBG*	11	00	3 / 6	4 / 14	0 / 8	3 / 18	2 / 4	8 / 10	6 / 16	XP_024854027.1:p.Leu238Pro
19	40977156	A	G	*KRT27*	00	11	2 / 6	6 / 14	1 / 8	9 / 18	1 / 4	6 / 10	7 / 16	ENSBTAP00000040718:p.Ser446Pro
19	40977827	C	A	*KRT27*	01	11	0 / 6	3 / 14	0 / 8	3 / 18	0 / 4	0 / 10	0 / 16	ENSBTAP00000040718:p.Gly404Cys
19	40978227	G	A	*KRT27*	01	00	0 / 6	0 / 14	2 / 8	1 / 18	0 / 4	0 / 10	1 / 16	ENSBTAP00000040718:p.Thr333Met
19	40982347	C	G	*KRT27*	01	00	0 / 6	2 / 14	2 / 8	3 / 18	0 / 4	0 / 10	2 / 14	ENSBTAP00000040718:p.Gly60Ala
20	31869993	G	A	*GHR*	11	00	6 / 6	14 / 14	8 / 8	18 / 18	4 / 4	10 / 10	16 / 16	ENSBTAP00000065441:p.Thr541Ile, ENSBTAP00000001758:p.Thr563Ile, ENSBTAP00000058520:p.Thr570Ile, ENSBTAP00000065610:p.Thr620Ile
20	39789981	C	T	*SLC45A2*	00	01	0 / 6	0 / 14	0 / 8	0 / 18	1 / 4	0 / 10	0 / 16	ENSBTAP00000024272:p.Leu64Phe
20	39792306	T	A	*SLC45A2*	00	01	1 / 6	4 / 14	2 / 8	2 / 18	1 / 4	2 / 10	4 / 16	ENSBTAP00000024272:p.Phe230Tyr
