# Supplementary material for: Genome of the extinct Gotland cattle breed
Source: BMC Genomics. 2025 Dec 3;26:1093. doi: 10.1186/s12864-025-12382-3 (PMC12690854; doi:10.1186/s12864-025-12382-3)
Supplement: Supplementary file 3 — Supplementary Figures [file 12864_2025_12382_MOESM3_ESM.pdf]

Genome of the extinct Gotland cattle breed

Fig. S1. Depth of coverage, Gotland cattle sample 1.
Fig. S2. Depth of coverage, Gotland cattle sample 2.
Fig. S3. Breadth of coverage, Gotland cattle sample 1.
Fig. S4. Breadth of coverage, Gotland cattle sample 2.
Fig. S5. Sensitivity analysis of principal component analysis.
Fig. S6. Model-based clustering with ADMIXTURE.
Fig. S7. mtDNA network.
Supplemental Table S1. Potential loss-of-function variants detected in candidate genes in Gotland cattle.
Supplemental Table S2. Missense variants detected in candidate genes in Gotland cattle.

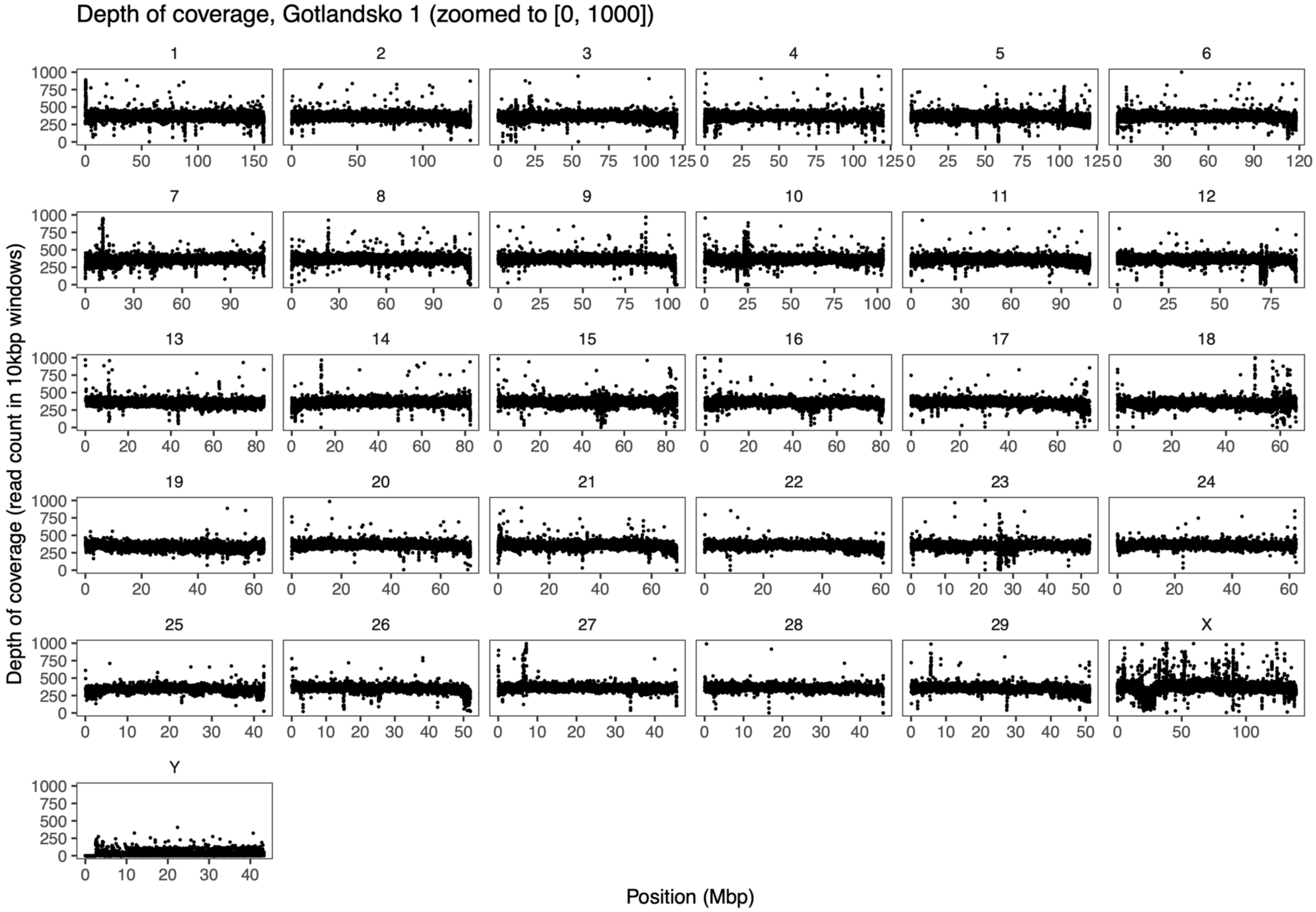

Fig. S1. Depth of coverage. Number of reads aligning in 10 kbp windows along the genome for Gotland cattle sample 1.

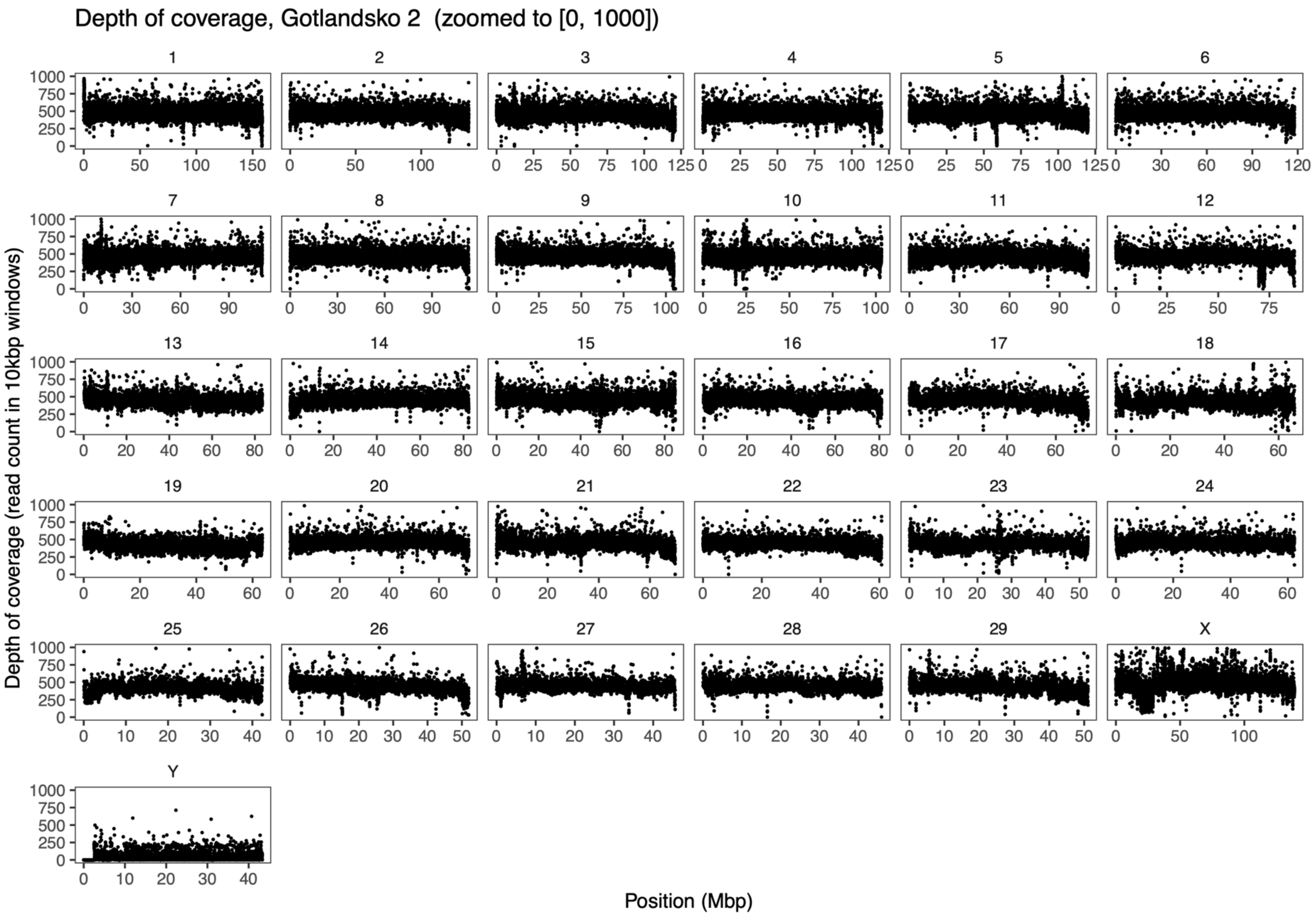

Fig. S2. Depth of coverage. Number of reads aligning in 10 kbp windows along the genome for Gotland cattle sample 2.

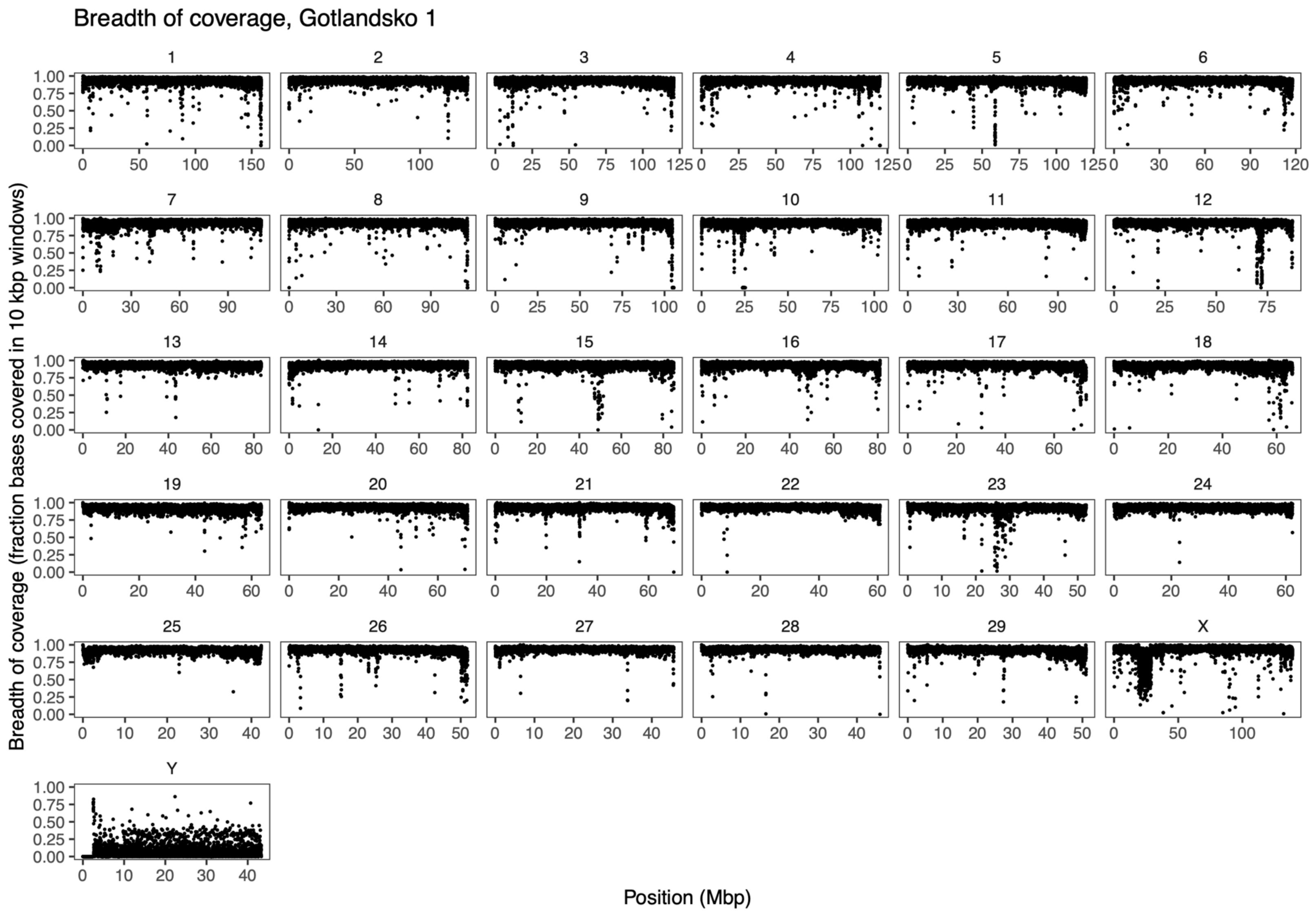

Fig. S3. Breadth of coverage. Fraction of bases covered by at least one read for 10 kbp windows along the genome for Gotland cattle sample 1.

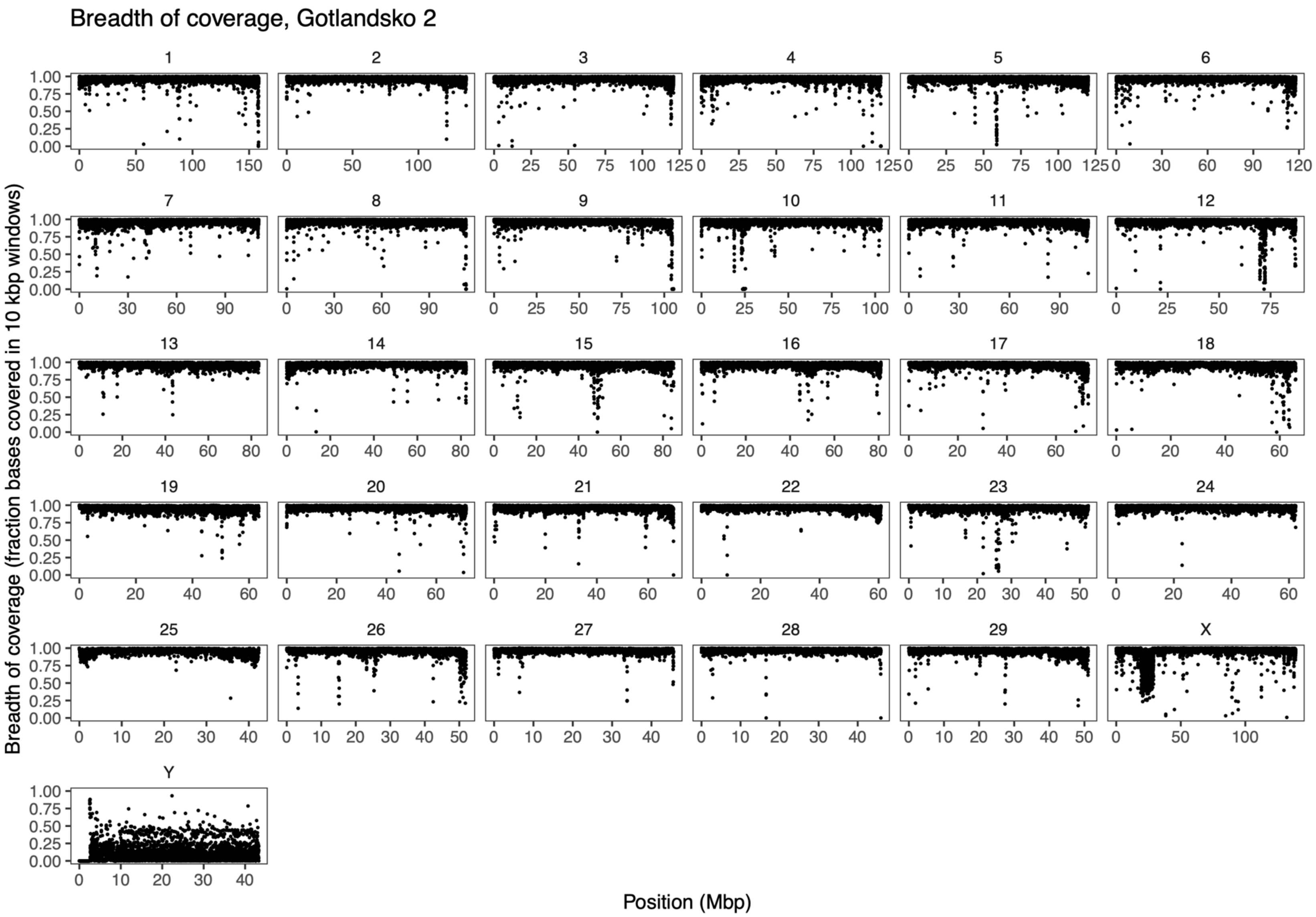

Fig. S4. Breadth of coverage. Fraction of bases covered by at least one read for 10 kbp windows along the genome for Gotland cattle sample 2.

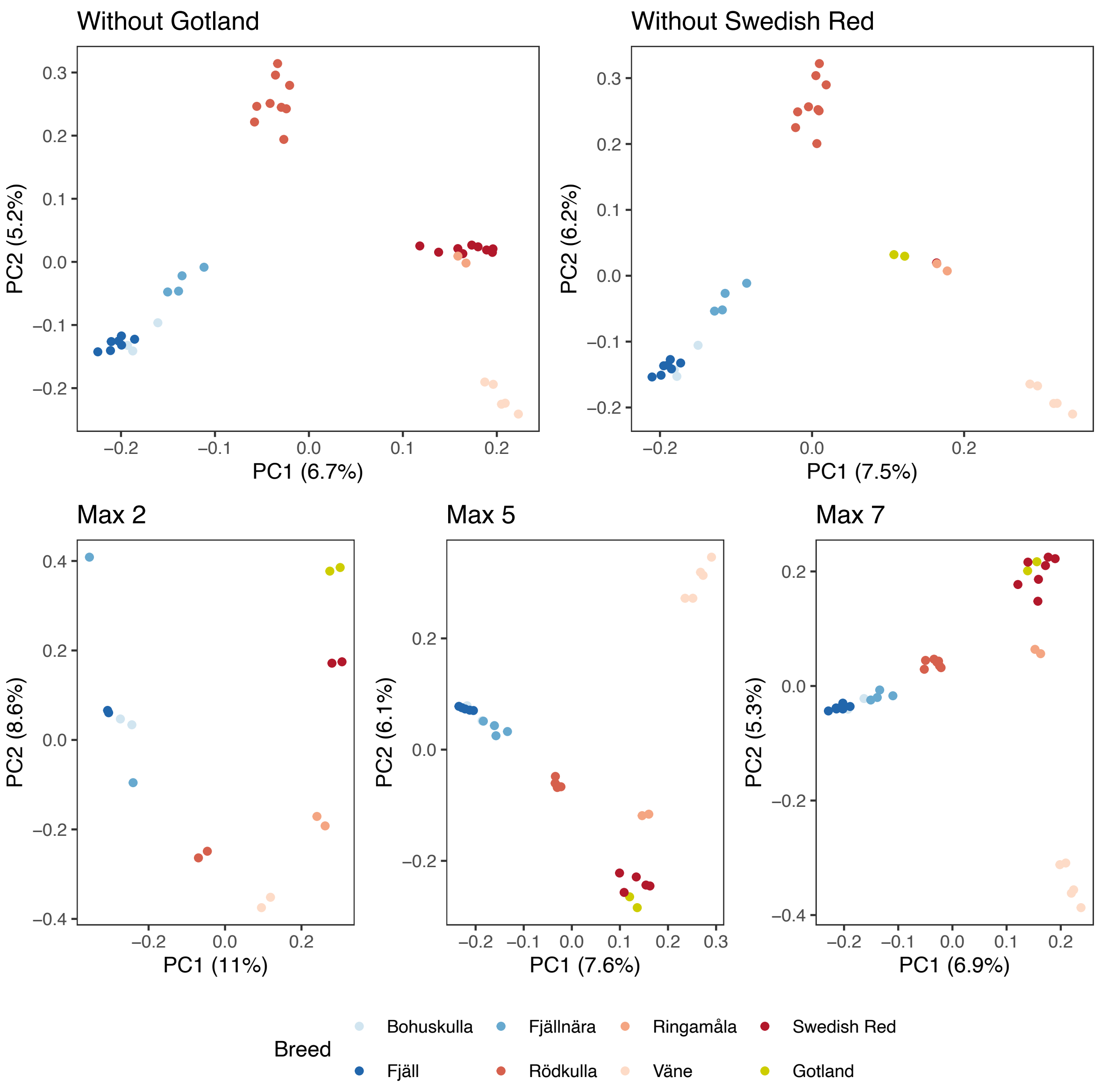

Fig. S5. Sensitivity analysis of principal component analysis. Plots of the first and second principal component from principal component analyses using subsets of the samples: excluding the Gotland cattle samples, excluding the Swedish Red samples, and subsetting the samples to include at most 2, 5 or 7 cattle in each group.

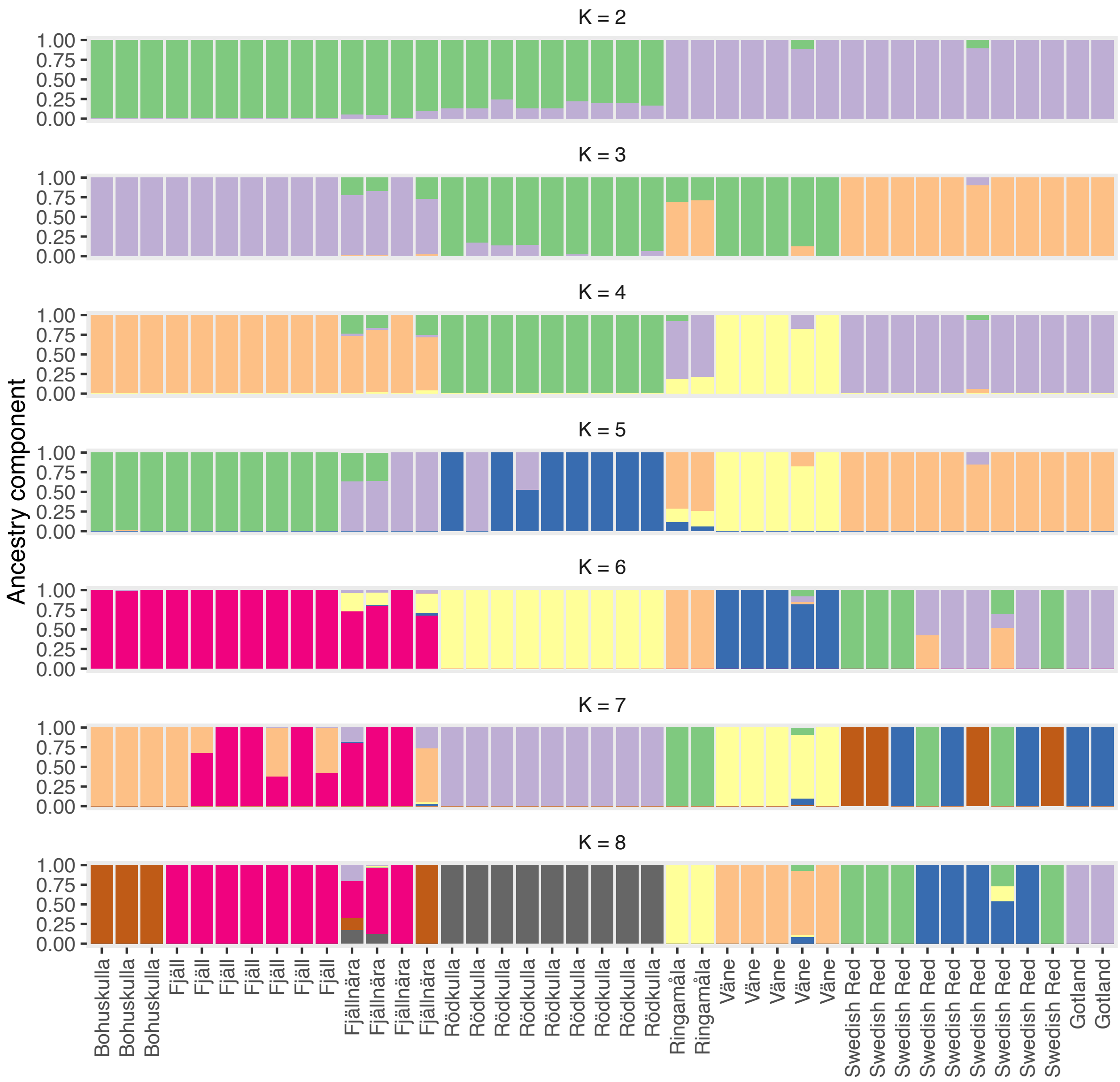

Fig. S6. Model-based clustering using ADMIXTURE. The colours of the bars show the contributions of the hypothetical ancestral populations to each animal's genome. Each bar is an animal, labelled by its breed. The panels show results for different values for the number of ancestral populations (K).

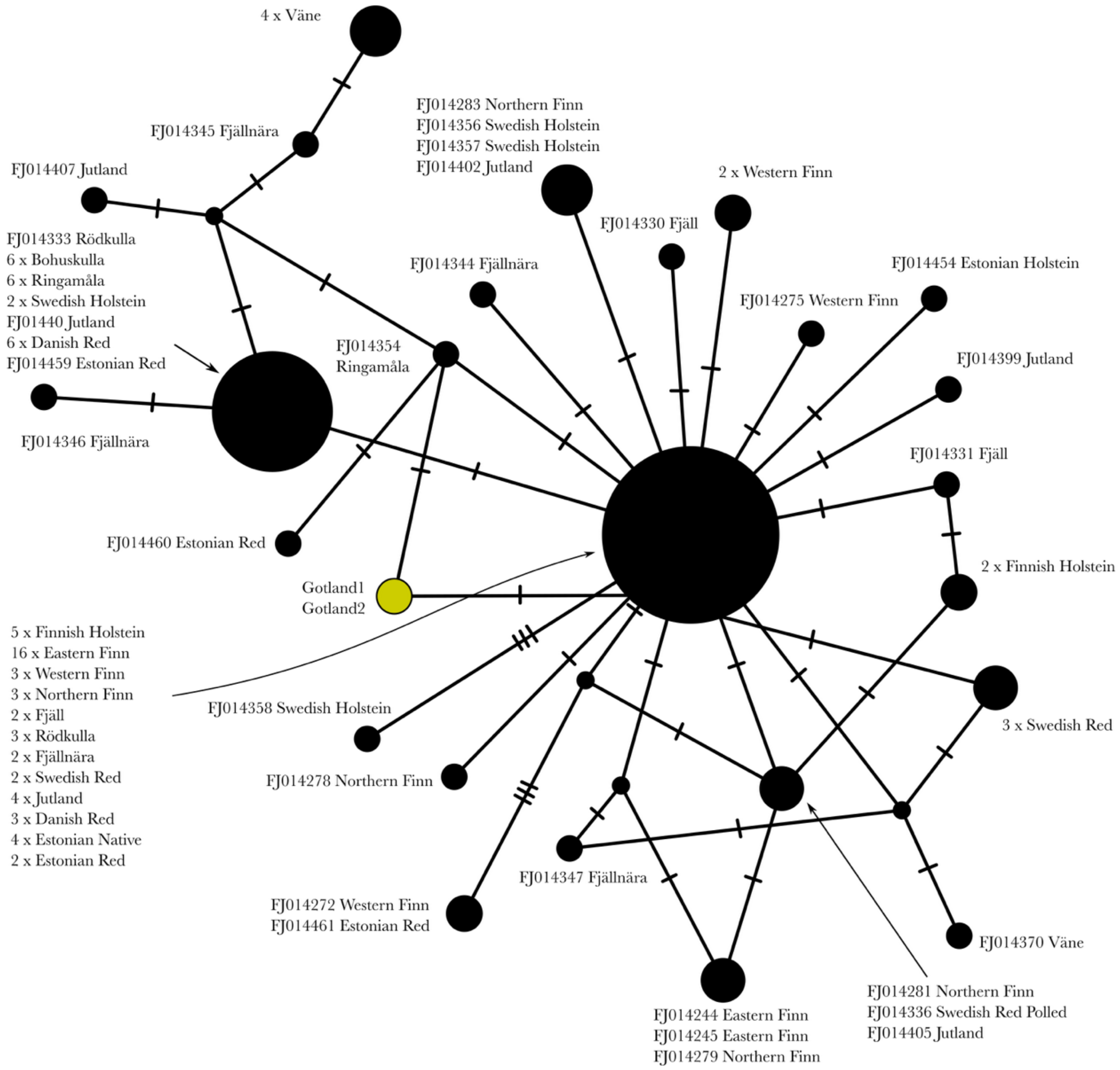

Fig. S7. mtDNA network with Gotland cattle compared to Nordic and Baltic mitochondrial D-loop sequences (Kantanen et al., 2009).
